# Older Adults Pursue More Autonomy During Pandemic: An Explanation by Social Obligation

**DOI:** 10.1093/geroni/igab046.730

**Published:** 2021-12-17

**Authors:** Hiu Ling Vivian Tsang, Chunyan Mai, Helene Fung

**Affiliations:** 1 Hong Kong Metropolitan University, The Chinese University of Hong Kong, Hong Kong; 2 The Chinese University of Hong Kong, Hong Kong, Hong Kong; 3 The Chinese University of Hong Kong, Shatin, N.T., Hong Kong, Hong Kong

## Abstract

Older adults are considered more vulnerable under the COVID-19 pandemic. Nevertheless, the pandemic also highlights the social obligation of all individuals, young and old. We investigated whether older adults pursued more autonomy during the pandemic than did middle-aged adults, and the moderating effect of perceived social obligation. One hundred and twenty-three Hong Kong citizens (62 females, Mage=60.59±13.28 years old) participated in this study in 2018 (before pandemic) and 2020 (during pandemic). Comparing these two waves, the results showed a larger increase of perceived importance of independence and autonomy among older adults than among middle-aged adults. Moreover, the age difference became stronger with a higher increase in expectation on social obligation, suggesting that the pandemic might make older adults feel more socially obligated to be independent and autonomous, so as not to be a burden on others. Future ageism-related studies should take the social obligation of older adults into consideration.

